# Differences in adoption of COVID-19 pandemic related preventive behaviour by viral load suppression status among people living with HIV during the first wave of the pandemic

**DOI:** 10.1186/s13104-023-06363-6

**Published:** 2023-05-25

**Authors:** Morenike Oluwatoyin Folayan, Roberto Ariel Abeldaño Zuñiga, Nourhan M. Aly, Passent Ellakany, Ifeoma E. Idigbe, Mohammed Jafer, Folake B. Lawal, Zumama Khalid, Joanne Lusher, Jorma I. Virtanen, Annie L Nguyen

**Affiliations:** 1 Mental Health and Wellness Study Group, Obafemi Awolowo University, Ile-Ife, Nigeria; 2 Department of Child Dental Health, Obafemi Awolowo University, Ile-Ife, Nigeria; 3 Faculty of Social Sciences, University of Helsinki, Helsinki, Finland; 4 Postgraduate Department, University of Sierra Sur, Oaxaca, Mexico; 5 Department of Pediatric Dentistry and Dental Public Health, Faculty of Dentistry, Alexandria University, Alexandria, Egypt; 6 Department of Substitutive Dental Sciences, College of Dentistry, Imam Abdulrahman Bin Faisal University, Dammam, Saudi Arabia; 7 Clinical Sciences Department, Nigerian Institute of Medical Research, Lagos, Nigeria; 8 Department of Preventive Dental Sciences, Faculty of Dentistry, Jazan University, Jazan, Saudi Arabia; 9 Department of Periodontology and Community Dentistry, University of Ibadan and University College Hospital, Ibadan, Nigeria; 10 Department of Health Sciences, University of Genova, Genova, GE 16132 Italy; 11 Regent’s University, London, UK; 12 Faculty of Medicine, University of Turku, Turku, Finland; 13 Department of Family Medicine, Keck School of Medicine, University of Southern California, Los Angeles, CA USA

**Keywords:** HIV, COVID-19, Preventive measures, Antiretroviral therapy

## Abstract

**Background:**

Adherence to antiretroviral therapy and COVID-19 preventive behaviours among people living with HIV during the pandemic has received little attention in the literature. To address this gap in knowledge, the present study assessed the associations between viral load, adherence to antiretroviral therapy and the use of COVID-19 prevention strategies during the first wave of the COVID-19 pandemic. This was a secondary analysis of data generated through an online survey recruiting participants from 152 countries. Complete data from 680 respondents living with HIV were extracted for this analysis.

**Results:**

The findings suggest that detectable viral load was associated with lower odds of wearing facemasks (AOR: 0.44; 95% CI:0.28–0.69; p < 0.01) and washing hands as often as recommended (AOR: 0.64; 95% CI: 0.42–0.97; p = 0.03). Also, adherence to the use of antiretroviral drugs was associated with lower odds of working remotely (AOR: 0.60; 95% CI: 0.38–0.94; p = 0.02). We found a complex relationship between HIV positive status biological parameters and adherence to COVID-19 preventive measures that may be partly explained by risk-taking behaviours. Further studies are needed to understand the reasons for the study findings.

## Introduction

Greater risk-taking attitudes and behaviour are commonly associated with poorer health outcomes [1]. Risk-takers typically place greater emphasis on immediate, usually gratifying, outcomes and less focus on longer-term outcomes, which may be positive but require a delay in experiencing the benefits [2–5]. Risk-takers value immediate rewards over distant rewards, with perceived value diminishing the further into the future it appears [6]. Referred to as delay-discounting, this is demonstrated when risk-takers choose smaller but more immediate rewards over larger but more distant rewards [7, 8].

Risk-taking is reflected in behaviours like high-risk sexual behaviour and substance use [9–14] and results from a combination of low-risk perception and high-risk propensity [15]. For example, low perception of risk for HIV is associated with engagement in activities such as having multiple sexual partners, early age of first sexual intercourse, unprotected sexual intercourse and inconsistent use of condoms, and not seeking treatment for sexually transmitted diseases [16, 17]. Low risk perception also inhibits the motivation to use and adhere to HIV prevention [18], delays the initiation of antiretroviral therapy for people living with HIV [19], and when on treatment, is associated with poor adherence to antiretroviral therapy [20]. Poor adherence to antiretroviral therapy is objectively assessed by viral load, or the number of copies of the HIV copies in one millilitre of blood. Similarly, perception of risk for COVID-19 can predict compliance with preventive behaviours and social distancing measures [21, 22]. Males, younger people and people with lower education status are more likely to have high-risk taking behaviours related to COVID-19 [15].

Because risk-taking is a behavioural expression of high-risk propensity [23], and this trait can manifest among people living with HIV as poor adherence to antiretroviral therapy, we posit that poor adherence will be associated with lower likelihood of COVID-19 prevention adoption. For people living with HIV, poor adherence to antiretroviral therapy leads to having detectable HIV viral loads [24]. The aim of this study therefore, was to explore these relationships by determining whether an association exists between viral load, adherence to antiretroviral treatment, and the use of COVID-19 prevention strategies.

## Main text

This was a secondary analysis of data generated through an online survey that recruited study participants from 152 countries between July and December 2020. The details of the parent study, including participants’ recruitment process [25, 26] and the tool used to collect the data [27], have been previously published. Ethical approval for the study was obtained from the Human Research Ethics Committee at the Institute of Public Health of the Obafemi Awolowo University Ile-Ife, Nigeria (HREC No: IPHOAU/12/1557). Additional ethical approvals were attained from India (D-1791-uz and D-1790-uz), Saudi Arabia (CODJU-2006 F), Brazil (CAAE N° 38423820.2.0000.0010) and United Kingdom (13,283/10,570). Study participants provided consent before participating in the online survey.

In the parent study, 904 respondents self-reported as being HIV positive. Of those, data from 680 (75.2%) respondents with complete responses for the dependent, independent, and confounding variables were extracted for this study. Respondents self-reported as being HIV positive by indicating the condition on a checklist of 27 medical ailments.

The dependent variables for this study were the use of COVID-19 prevention strategies: physical distancing, wearing of facemasks, hands washing, and working remotely. Respondents were asked if they adopted any of these behaviour for use during the pandemic. Respondents could select more than one item if they adopted multiple preventive behaviours during the pandemic.

The independent variable were the viral load and adherence to antiretroviral treatment. Participants selected response to questions about viral load and adherence: (1) What is your HIV viral load (undetectable, detectable and do not know), and (2) Some people find that they sometimes forget to take their medications to manage their HIV. Did you miss any of your HIV medications during COVID-19 (Yes, No)? Participants who noted they did not know their HIV status were excluded from analyses;

Confounding variables considered included age at last birthday [28, 29], sex at birth (male, female, intersex, no response) [30, 31] educational status (No formal education, primary level, secondary level and tertiary level) and self-report of depression [32, 33]. Depression was assessed by asking respondents to indicate if they had experienced any of the 10 listed emotions during the pandemic, one of which was depression. Respondents who did not check the box were categorised as not having experienced depression during the pandemic. Depression has been shown to be associated with HIV infection and has increased in prevalence during the COVID-19 pandemic [34–37].

Four multivariable logistic regression analyses were conducted to determine the associations between the dependent and independent variables after adjusting for the confounding variables. Adjusted odds ratios (AOR) and 95% confidence intervals (CI) were calculated. Statistical significance was set at 5%.

## Results

Of the 680 respondents living with HIV in this study, 459 (67.5%) kept physical distance, 581 (85.4%) wore face masks, 548 (80.6%) washed hands as often as recommended, and 140 (20.6%) worked remotely during the pandemic. As can be seen in Tables 1 and 488 (71.8%) were virally suppressed, 529 (77.8%) adhered to the use of the antiretrovirals and 128 (18.8%) were depressed during the lockdown.

People living with HIV with a detectable viral load had lower odds of physical distancing, wearing facemasks, washing hands as often as recommended and working remotely. The association was, however, only significant for wearing facemasks (AOR: 0.44; 95% CI:0.28–0.69; p < 0.01) and washing hands as often as recommended (AOR: 0.64; 95% CI: 0.42–0.97; p = 0.03) during the pandemic. Also, people living with HIV who adhered to the use of the antiretroviral drugs had statistically significant for lower odds of working remotely (AOR: 0.60; 95% CI: 0.38–0.94; p = 0.02).

**Table 1:** Association between viral load, antiretroviral adherence profile and the adoption of COVID-19 pandemic preventive behaviour status among PLHIV during the first wave of the pandemic (N = 680)

Variables	Total N = 680 n (%)	Physical distancing			Wearing face masks			Washing hands as often as recommended			Work remotely		
		No 221(32.5%) n (%)	Yes 459(67.5%) n (%)	AOR; 95% CI; p value	No 99(14.6%) n (%)	Yes 581(85.4%) n (%)	AOR; 95% CI; p value	No 132(19.4%) n (%)	Yes 548(80.6%) n (%)	AOR; 95% CI; p value	No 540(79.4%) n (%)	Yes 140 (20.6) n (%)	AOR; 95% CI; p value
**Viral load**													
Undetectable Detectable	488 (71.8) 192 (28.2)	157 (32.2) 64 (33.3)	331 (67.8) 128 (66.7)	1 1.06; 0.72–1.55; p = 0.74	56 (11.5) 43 (22.4)	432 (88.5) 149 (77.6)	1 0.44; 0.28–0.69;p < 0.01	86 (17.6) 46 (24.0)	402 (82.4) 146 (76.0)	1 0.64; 0.42–0.97;p = 0.03	389 (79.7) 151 (78.6)	99 (20.3) 41 (21.4)	1 0.99; 0.64–1.53; p = 0.98
**Adherence**													
**to ART** No Yes	151 (22.2) 529 (77.8)	53 (35.1) 168 (31.8)	98 (64.9) 361 (68.2)	1 1.04; 0.69–1.58; p = 0.83	23 (15.2) 76 (14.4)	128 (84.8) 453 (85.6)	1 0.99; 0.57–1.71;p = 0.98	30 (19.9) 102 (19.3)	121 (80.1) 427 (80.7)	1 1.11; 0.69–1.81;p = 0.65	107 (70.9) 433 (81.9)	44 (29.1) 96 (18.1)	1 0.60; 0.38–0.94; p = 0.02
**Age**	39.9 (11.3)	39.1 (11.6)	40.3 (11.2)	1.01; 1.00-1.03; p = 0.38	40.9 (11.3)	39.7 (11.3)	0.99; 0.97-1.00;p = 0.51	39.3 (10.1)	40.0 (11.6)	1.07; 0.98–1.02; p = 0.46	39.6 (11.6)	40.8 (10.3)	1.01; 0.99–1.03; p = 0.17
**Sex**													
Male Female Intersex Decline to answer	277 (40.8) 395 (58.1) 3 (0.4) 5 (0.7)	89 (32.1) 129 (32.7) 2 (66.7) 1 (20.0)	188 (67.9) 266 (67.3) 1 (33.3) 4 (80.0)	1 0.97; 0.69–1.38; p = 0.89 0.67; 0.04–9.71; p = 0.77 1.72; 0.18–15.82; p = 0.62	40 (14.4) 56 (14.2) 1 (33.3) 2 (40.0)	237 (85.6) 339 (85.8) 2 (66.7) 3 (60.0)	1 0.94; 0.59–1.48;p = 0.80 0.56; 0.03–8.87;p = 0.68 0.20; 0.03–1.29;p = 0.09	48 (17.3) 82 (20.8) 1 (33.3) 1 (20.0)	229 (82.7) 313 (79.2) 2 (66.7) 4 (80.0)	1 0.81; 0.54–1.22;p = 0.32 0.53; 0.03–7.87;p = 0.64 0.96; 0.10–8.94;p = 0.97	217 (78.3) 316 (80.0) 2 (66.7) 5 (100.0)	60 (21.7) 79 (20.0) 1 (33.3) 0 (0.0)	1 0.93; 0.63–1.39; p = 0.75 3.30; 0.22–49.46; p = 0.38 Not calculated (p = 0.99)
**Educational level**													
No formal Primary Secondary Tertiary	24 (3.5) 57 (8.4) 239 (35.1) 360 (52.9)	20 (83.3) 30 (52.6) 72 (30.1) 99 (27.5)	4 (16.7) 27 (47.4) 167 (69.9) 261 (72.5)	0.66; 0.02–0.20; p < 0.01 0.34; 0.19–0.60; p < 0.01 0.94; 0.65–1.36; p = 0.75 1	8 (33.3) 14 (24.6) 31 (13.0) 46 (12.8)	16 (66.7) 43 (75.4) 208 (87.0) 314 (87.2)	0,30; 0,11 − 0,79; p = 0,01 0,43; 0,21 − 0,87; p = 0,01 0,96; 0,58 − 1,59; p = 0,89 1	6 (25.0) 12 (21.1) 51 (21.3) 63 (17.5)	18 (75.0) 45 (78.9) 188 (78.7) 297 (82.5)	0,49; 0,17 − 1,39; p = 0,18 0,78; 0,38 − 1,60; p = 0,50 0,82; 0,53 − 1,26; p = 0,37 1	21 (87.5) 53 (93.0) 203 (84.9) 263 (73.1)	3 (12.5) 4 (7.0) 36 (15.1) 97 (26.9)	0,24; 0,06 − 0,89; p = 0,03 0,19; 0,06 − 0,55; p < 0,01 0,53; 0,34 − 0,82; p < 0,001 1
**Depression**													
No Yes	552 (81.2) 128 (18.8)	177 (32.1) 44 (34.4)	375 (67.9) 84 (65.6)	1 1.09; 0.70–1.69; p = 0.70	86 (15.6) 13 (10.2)	466 (84.4) 115 (89.8)	1 1.90; 0.99–3.68;p = 0.05	124 (22.5) 8 (6.3)	428 (77.5) 120 (93.7)	1 4.80; 2.24–10.27;p < 0.01	453 (82.1) 87 (68.0)	99 (17.9) 41 (32.0)	1 2.10; 1.33–3.31; p < 0.01

*ART – Antiretroviral therapy

## Discussion

The results partly support our study hypothesis that poor adherence to antiretroviral therapy (reflected by detectable viral load) will be positively associated with poorer use of COVID-19 preventive behaviours. The study findings suggest that people living with HIV who had detectable viral load and who poorly adhered to antiretroviral therapy were less likely to adhere to COVID-19 preventive measures though this was only significant for wearing facemasks and washing hands as often as recommended. On the contrary, people living with HIV who adhered to antiretroviral therapy were less like to working remotely.

Risk-taking propensity has been shown to be directly associated with poor mask-wearing and physical distancing behaviours [38]. Risk-taking has also been associated with increased risk for HIV infection [39], and lower engagement in sexual risk behaviours [40]. Study findings suggest that risk-taking related to HIV treatment may reinforce risk-taking for COVID-19 prevention [41]. However, on the contrary, we observed that people living with HIV who adhere to the use of antiretroviral therapy were less likely to work remotely. This result may reflect a compensatory behaviour whereby the regular wearing of facemasks and handwashing may be considered enough precautionary measure to outweigh the risk for contracting infection, and address the need to reduce self-isolation (working remotely). This postulation ggives credence to a risk compensation hypothesis [42].

The risk compensation hypothesis implies that persons experiencing a real or perceived change in the riskiness of an activity will alter their consumption of that activity to obtain a preferred combination of risk and reward [43]. We hypothesis that people living with HIV who adhere to the use of antiretroviral therapy, wear facemasks, wash hands as is regularly required and adhere to physical distancing measures may feel this is safe enough interventions to reduce their risk for contracting COVID-19 if they do not work remotely. A decision not to work remotely may be connected with a decision to avoid social isolation, a risk factor for loneliness [44] and a critical risk factor for poor adherence to ART [45] and mortality for people living with HIV [46].

Risk-taking and non-adherence with COVID-19 preventive measures may be an active or passive risk-taking behaviour which are associated with different personal tendencies [47]. The failure to wear face masks and not to wash hands as recommended may be a result of passive risk-taking behaviour while the decision not to work remotely results from active risk-taking behaviour [47]. Personality traits like having more self-control, reduces passive risk-taking because future consequences of their actions are taken into account when making decisions about taking risks [48]. Active risk-takers take the future into perspectives when taking decisions about risk [47].

Our prior study had indicated that people living with HIV were more likely not to keep physical distance, isolated/quarantined and worked remotely when compared with people not living with HIV suggesting that the community may have concerns with social isolation and thus, will take actions to promote social engagement [49]. The current study finding however, suggests that individual personality traits linked to risk-taking behaviours may mediate the link between the biological profile of people living with HIV (viral load and adherence to use of antiretroviral drugs) and poor compliance with COVID-19 preventive measures. Personality traits linked to passive risk-taking may mediate the association between wearing of facemasks and not washing hands as recommended and viral load status; and the traits associated with active risk-taking may mediate the association between working remotely and adherence with use of antiretroviral therapy. It is therefore important that people living with HIV are counselled on-on-one to understand decision-taking about the use of COVID-19 preventive measures, and supported to address concerns and risk.

We observe a complex relationship exists between HIV status and the use of COVID-19 preventive measures that can be partially explained by individual’s risk-taking decisions. We postulate that people living with HIV who have passive risk-taking propensity are less likely to adhere to COVID-19 preventive measures. However, irrespective of the risk-taking propensity, people living with HIV are less likely to adopt COVID-19 preventive measures that promote social isolation and its associated risk for their long-term health and wellbeing through a risk compensation decision-making process. This postulation needs to be tested.

### Limitations

This was a cross-sectional study and so cause-effect deductions cannot be made. Also, the data were collected using a non-probability sampling methods which limits the generalisability of the study findings. Also, the study had no access to data on the socioeconomic status and changes in socioeconomic status of participants during the pandemic. In addition, we made deductions about the roles of risk-taking behaviours in moderating the findings. Discussions about risk-taking behaviours need to be taken with caution as the risk-taking behaviour of study participants was not measured objectively. HIV status was also self-reported with a risk for underestimation of the HIV positive status of respondents (about 15% of the global population do not know their HIV status) [50]. Also, the study did not account for the possible biases that the socio-economic status of respondents could introduce. The findings reported here needs further exploration.

## Data Availability

The datasets used and/or analysed during the current study are available from the corresponding author on reasonable request.

## Abbreviations

AOR: Adjusted Odds Ratio
ART: Antiretroviral Therapy
CI: Confidence Interval
COVID-19: Coronavirus infectious disease 2019
HIV: Human Immunodeficiency Virus
