# Correction: Differences in adoption of COVID-19 pandemic related preventive behaviour by viral load suppression status among people living with HIV during the first wave of the pandemic

**DOI:** 10.1186/s13104-023-06419-7

**Published:** 2023-08-22

**Authors:** Morenike Oluwatoyin Folayan, Roberto Ariel Abeldaño Zuñiga, Nourhan M. Aly, Passent Ellakany, Ifeoma E. Idigbe, Mohammed Jafer, Folake B. Lawal, Zumama Khalid, Joanne Lusher, Jorma I. Virtanen, Annie L. Nguyen

**Affiliations:** 1 Mental Health and Wellness Study Group, Obafemi Awolowo University, Ile-Ife, Nigeria; 2 Department of Child Dental Health, Obafemi Awolowo University, Ile-Ife, Nigeria; 3 Faculty of Social Sciences, University of Helsinki, Helsinki, Finland; 4 Postgraduate Department, University of Sierra Sur, Oaxaca, Mexico; 5 Department of Pediatric Dentistry and Dental Public Health, Faculty of Dentistry, Alexandria University, Alexandria, Egypt; 6 Department of Substitutive Dental Sciences, College of Dentistry, Imam Abdulrahman Bin Faisal University, Dammam, Saudi Arabia; 7 Clinical Sciences Department, Nigerian Institute of Medical Research, Lagos, Nigeria; 8 Department of Preventive Dental Sciences, Faculty of Dentistry, Jazan University, Jazan, Saudi Arabia; 9 Department of Periodontology and Community Dentistry, University of Ibadan and University College Hospital, Ibadan, Nigeria; 10 Department of Health Sciences, University of Genova, Genoa, GE 16132 Italy; 11 Regent’s University, London, UK; 12 Faculty of Medicine, University of Turku, Turku, Finland; 13 Department of Family Medicine, Keck School of Medicine, University of Southern California, Los Angeles, CA USA

**Correction: BMC Research Notes (2023) 16:90** 10.1186/s13104-023-06363-6

Following the publication of the original article [1] it was brought to our attention that Table 1 contained some errors inadvertently introduced during typesetting.

The correct Table 1 is shown in this erratum:

**Table 1:** Association between viral load, antiretroviral adherence profile and the adoption of COVID-19 pandemic preventive behaviour status among PLHIV during the first wave of the pandemic (N = 680)

Variables	Total	Physical distancing			Face mask			Handwashing			Work remotely		
		No	Yes	AOR; 95% CI; p value	No	Yes	AOR; 95% CI; p value	No	Yes	AOR; 95% CI; p value	No	Yes	AOR; 95% CI; p value
	N = 680	221 (32.5%)	459 (67.5%)		99 (14.6%)	581 (85.4%)		132 (19.4%)	548 (80.6%)		540 (79.4%)	140 (20.6)	
	n (%)	n (%)	n (%)		n (%)	n (%)		n (%)	n (%)		n (%)	n (%)	
Viral load													
Undetectable	488 (71.8)	157 (32.2)	331 (67.8)	1	56 (11.5)	432 (88.5)	1	86 (17.6)	402 (82.4)	1	389 (79.7)	99 (20.3)	1
Detectable	192 (28.2)	64 (33.3)	128 (66.7)	1.06; 0.72–1.55; p = 0.74	43 (22.4)	149 (77.6)	0.44; 0.28–0.69; p < 0.01	46 (24.0)	146 (76.0)	0.64; 0.42–0.97; p = 0.03	151 (78.6)	41 (21.4)	0.99; 0.64–1.53; p = 0.98
Adherence to ART													
No	151 (22.2)	53 (35.1)	98 (64.9)	1	23 (15.2)	128 (84.8)	1	30 (19.9)	121 (80.1)	1	107 (70.9)	44 (29.1)	1
Yes	529 (77.8)	168 (31.8)	361 (68.2)	1.04; 0.69–1.58; p = 0.83	76 (14.4)	453 (85.6)	0.99; 0.57–0.71; p = 0.98	102 (19.3)	427 (80.7)	1.11; 0.69–1.81; p = 0.65	433 (81.9)	96 (18.1)	0.60; 0.38–0.94; p = 0.02
Age	39.9(11.3)	39.1 (11.6)	40.3 (11.2)	1.01; 1.00–1.03; p = 0.38	40.9 (11.3)	39.7 (11.3)	0.99; 0.97–1.00; p = 0.51	39.3 (10.1)	40.0 (11.6)	1.07; 0.98–1.02; p = 0.46	39.6 (11.6)	40.8 (10.3)	1.01; 0.99–1.03; p = 0.17
Sex													
Male	277 (40.8)	89 (32.1)	188 (67.9)	1	40 (14.4)	237 (85.6)	1	48 (17.3)	229 (82.7)	1	217 (78.3)	60 (21.7)	1
Female	395 (58.1)	129 (32.7)	266 (67.3)	0.97; 0.69–1.38; p = 0.89	56 (14.2)	339 (85.8)	0.94; 0.59–0.48; p = 0.80	82 (20.8)	313 (79.2)	0.81; 0.54–1.22; p = 0.32	316 (80.0)	79 (20.0)	0.93; 0.63–1.39; p = 0.75
Intersex	3 (0.4)	2 (66.7)	1 (33.3)	0.67; 0.04–9.71; p = 0.77	1 (33.3)	2 (66.7)	0.56; 0.03–0.87; p = 0.68	1 (33.3)	2 (66.7)	0.53; 0.03–7.87; p = 0.64	2 (66.7)	1 (33.3)	3.30; 0.22–49.46; p = 0.38
Decline to answer	5 (0.7)	1 (20.0)	4 (80.0)	1.72; 0.18–15.82; p = 0.62	2 (40.0)	3 (60.0)	0.20; 0.03–0.29; p = 0.09	1 (20.0)	4 (80.0)	0.96; 0.10–8.94; p = 0.97	5 (100.0)	0 (0.0)	Not calculated (p = 0.99)
Educational level													
No formal	24 (3.5)	20 (83.3)	4 (16.7)	0.66; 0.02–0.20; p < 0.01	8 (33.3)	16 (66.7)	0.30; 0.11–0.79; p = 0.01	6 (25.0)	18 (75.0)	0.49; 0.17–1.39; p = 0.18	21 (87.5)	3 (12.5)	0.24; 0.06–0.89; p = 0.03
Primary	57 (8.4)	30 (52.6)	27 (47.4)	0.34; 0.19–0.60; p < 0.01	14 (24.6)	43 (75.4)	0.43; 0.21–0.87; p = 0.01	12 (21.1)	45 (78.9)	0.78; 0.38–1.60; p = 0.50	53 (93.0)	4 (7.0)	0.19; 0.06–0.55; p < 0.01
Secondary	239 (35.1)	72 (30.1)	167 (69.9)	0.94; 0.65–1.36; p = 0.75	31 (13.0)	208 (87.0)	0.96; 0.58–1.59; p = 0.89	51 (21.3)	188 (78.7)	0.82; 0.53–1.26; p = 0.37	203 (84.9)	36 (15.1)	0.53; 0.34–0.82; p < 0.001
Tertiary	360 (52.9)	99 (27.5)	261 (72.5)	1	46 (12.8)	314 (87.2)	1	63 (17.5)	297 (82.5)	1	263 (73.1)	97 (26.9)	1
Depression													
No	52 (81.2)	177 (32.1)	375 (67.9)	1	86 (15.6)	466 (84.4)	1	124 (22.5)	428 (77.5)	1	453 (82.1)	99 (17.9)	1
Yes	128 (18.8)	44 (34.4)	84 (65.6)	1.09; 0.70–1.69; p = 0.70	13 (10.2)	115 (89.8)	1.90; 0.99–3.68; p = 0.05	8 (6.3)	120 (93.7)	4.80; 2.24–10.27; p < 0.01	87 (68.0)	41 (32.0)	2.10; 1.33–3.31; p < 0.01

*ART* antiretroviral therapy

The original article has been corrected.
